# Rare growth pattern of a solitary cystic lung metastasis from colon cancer: a case report

**DOI:** 10.1002/rcr2.474

**Published:** 2019-08-21

**Authors:** Keisuke Eguchi, Takahiro Nakajima, Takeshi Terashima, Aya Sasaki, Hirotoshi Hasegawa, Junichi Matsui

**Affiliations:** ^1^ Department of Surgery Tokyo Dental College Ichikawa General Hospital Chiba Japan; ^2^ Department of Respiratory Medicine Tokyo Dental College Ichikawa General Hospital Chiba Japan; ^3^ Department of Pathology and Laboratory Medicine Tokyo Dental College Ichikawa General Hospital Chiba Japan

**Keywords:** Cystic lung metastasis, double cancer, primary lung cancer, sigmoid colon cancer, video‐assisted thoracoscopic surgery

## Abstract

An 82‐year‐old male, who had undergone sigmoid colon cancer surgery (at the age of 78 years) and primary lung cancer surgery (at the age of 81 years), was found to have a cavitating lesion in the left lower lobe on chest computed tomography (CT). A chest CT that had been performed just before the primary lung cancer surgery revealed a small thin‐walled cyst at the same site at which the cavity was detected in the current CT. Bronchoscopic examination revealed no evidence of malignancy. A follow‐up chest CT performed 5 months later revealed that the lesion had grown and that the cyst contained a well‐defined lobular nodule. Video‐assisted thoracoscopic left basal segmentectomy was performed. The histopathological diagnosis was metastasis from colon cancer. We report this unusual case in which a pulmonary metastasis changed over time from a cystic lesion to a nodular lesion.

## Introduction

Pulmonary cystic metastasis (PCM) from colon cancer is rare; furthermore, in most cases, the lesions are multiple and unresectable. We report a surgical case of a solitary pulmonary metastasis in which the lesion evolved and enlarged from a cyst to a solid lesion with time.

## Case Report

**Figure 1:**
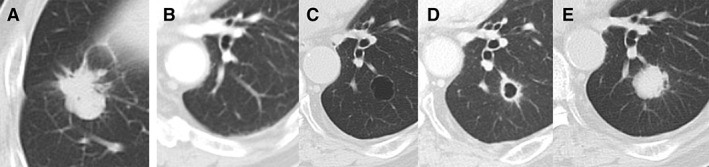
Chest computed tomography (CT) findings. (A) An irregular nodular opacity is detected in the right lower lobe, which was diagnosed as a primary lung cancer. (B) No abnormal finding in the left lower lobe is seen on the chest CT performed one year before the CT shown in (A). (C) At the same time of the CT examination of (A), a thin‐walled cyst was detected in the left lower lobe. (D) The cyst wall became thicker as seen in the image obtained one year after the lung cancer surgery. (E) In the CT obtained five months after the CT shown in (D), a nodular shadow is identified in the left lower lobe at the same site as the cavity lesion.

**Figure 2:**
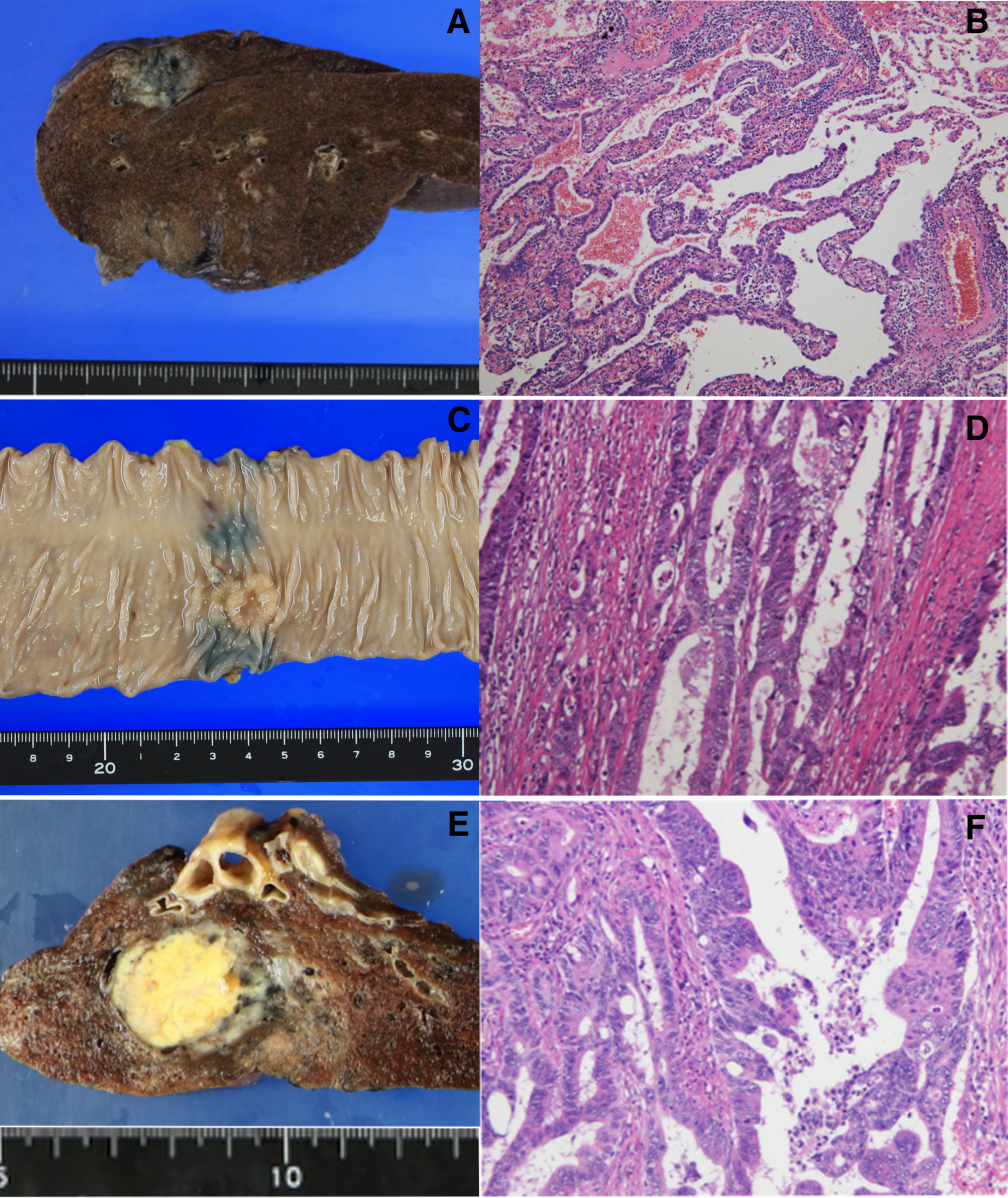
Macroscopic appearance and microscopic (haematoxylin–eosin staining) findings: (A) foci of necrosis, a central scar and anthracotic pigmentation was observed in a cross section of the primary lung cancer; (B) the histological diagnosis of the primary lung cancer was acinar adenocarcinoma; (C) a circumferential type‐2 tumour was seen in the resected sigmoid colon specimen; (D) the sigmoid colon tumour was diagnosed histologically as a moderately differentiated tubular adenocarcinoma; (E) a cross section of the tumour of the left lung revealed expansive growth, and necrosis in the greater part of the tumour; (F) the tumour in the lower lobe of the left lung was diagnosed as a metastatic lung tumour from the sigmoid colon cancer.

An 82‐year‐old male who had undergone sigmoidectomy for colon cancer three years ago was diagnosed as having primary lung adenocarcinoma (Fig. 1A), and underwent video‐assisted right lower lobectomy with lymph node dissection. The histopathological diagnosis was moderately differentiated tubular adenocarcinoma of the sigmoid colon (Fig. 2C, D), whereas the lung lesion was diagnosed as a primary acinar adenocarcinoma of the lung (Fig. 2A, B). Follow‐up chest computed tomography (CT) one year later showed a thick‐walled cavity (wall thickness >4 mm 1) in the lower lobe of the left lung (Fig. 1D). A retrospective review of the chest CT images obtained just before the primary lung cancer surgery a year previously revealed a thin‐walled cyst (wall thickness <2 mm 1) in the same location as that of the thick‐walled cavity in the current CT images (Fig. 1C). Furthermore, the lesion was not detectable in the CT obtained a year before the surgery (Fig. 1B). The serum carcinoembryonic antigen (CEA) level had remained within normal limits, except for an elevated value (7.5 ng/mL) detected once, prior to the primary lung cancer surgery. A 18F‐fluorodeoxyglucose positron emission tomography‐CT showed elevated uptake (SUVmax, 1.7) in a thick‐walled cavity measuring 1.6 cm in diameter in the lower lobe of the left lung. Bronchoscopy revealed no endoluminal lesion and transbronchial biopsy was negative for malignancy. However, in a follow‐up chest CT obtained five months later, the lesion had changed from a cystic lesion to a nodular lesion of 1.9 cm in diameter (Fig. 1E). It was suspected that a malignant pulmonary tumour or aspergilloma had grown in the pulmonary cyst. Video‐assisted thoracoscopic basal segmentectomy of the left lower lobe was performed, considering both the need for complete tumour resection and preservation of the post‐operative respiratory function. Histopathologically, the resected lung tumour was similar to the sigmoid colon cancer, and was therefore diagnosed as a metastasis from the colon cancer (Fig. 2E, F). The post‐operative course was uneventful. The patient did not receive post‐operative chemotherapy, and has lived without any recurrence of the tumour for 32 months after the last pulmonary resection.

## Discussion

The unique and complex characteristics of the clinical course of this patient were as follows: the patient developed a solitary PCM, which was surgically resectable; the pulmonary lesion increased in size, and its appearance was changed from a thin‐walled cyst to a thick‐walled cavity and then to a nodule; histopathology confirmed the tumour as a PCM from colon cancer in the patient who had been diagnosed as a case of metachronous colon and lung cancer.

It is clinically important to distinguish whether pulmonary cysts are malignant or non‐malignant (e.g. infectious). Primary lung cancer and pulmonary metastases are also known to rarely occur as thin‐wall cystic pulmonary lesions 2. Therefore, the difficulty in its detection often causes delayed diagnosis or misdiagnosis. Cavitation detectable by CT has been reported in up to 22% of all cases of primary lung cancer; however, occurrence of primary lung cancer as a thin‐walled cyst was unusual. Moreover, pulmonary metastases occur less frequently as thin‐walled cysts as compared to primary lung cancer 3.

Multifocal/diffuse cysts can occur in cases of lymphoid interstitial pneumonia, Birt‐Hogg‐Dubé syndrome, tracheobronchial papillomatosis, or primary and metastatic cancers 1. Distinguishing multiple PCM from benign bullae is often easy when many solid tumour nodules are also present along with multiple PCMs in the same lung 4, 5; on the other hand, it is difficult to distinguish a solitary PCM from a solitary pulmonary cyst, which may be an age‐related phenomenon or may be a remnant of prior trauma or infection 1. Presence of multiple PCMs is usually a contraindication for complete resection; however, solitary cystic lesions could be treated by surgical resection. In the present case, the ball‐valve phenomenon associated with the endobronchial tumour might have caused the cyst formation primarily, and the tumour might have grown secondarily in the cyst.

Because of the tendency of the tumour to increase in size with time, we presumed it to be a malignant tumour at first. Left lower lobectomy would have had to be selected if it were a second primary lung cancer. However, we avoided it so as to preserve the pulmonary function, because the patient was old and had already undergone right lower lobectomy. Wedge resection would have been suitable in the case of metastatic tumour; however, it was difficult anatomically. Therefore, we thought that segmentectomy was the most suitable for this patient, irrespective of whether the tumour was a second primary lung cancer or a metastatic lung tumour. To obtain more correct pre‐operative information about the tumour in this case, we should have performed CT‐guided biopsy.

The possibility that the tumour was primary enteric adenocarcinoma of the lung still remains. However, since the patient had the history of sigmoid colon cancer, we made the diagnosis of metastatic colon cancer. It is important that pulmonary metastasis is considered in the differential diagnosis in patients with a past history of malignant tumour presenting with solitary or multiple pulmonary cysts.

The patient has lived without any recurrence of the tumour for 32 months after the last pulmonary resection. However, careful post‐operative follow‐up is necessary to detect any new metastatic lesion.

### Disclosure Statement

Appropriate written informed consent was obtained for publication of this case report and accompanying images.

## References

[1] Raoof S , Bondalapati P , Vydyula R , et al. 2016 Cystic lung disease: algorithmic approach. Chest 150:945–965. 27180915 10.1016/j.chest.2016.04.026 PMC7534033

[2] Woodring JH , Fried AM , and Chuang VP . 1980 Solitary cavities of the lung: diagnostic implications of cavity wall thickness. AJR Am. J. Roentgenol. 135:1269–1271. 6779538 10.2214/ajr.135.6.1269

[3] Gadkowski LB , and Stout JE . 2008 Cavitary pulmonary disease. Clin. Microbiol. Rev. 21:305–333. 18400799 10.1128/CMR.00060-07 PMC2292573

[4] Sagar D , and Adeni A . 2018 Unusual cystic lung metastasis. BMJ Case Rep. 10.1136/bcr-2018-224648. PMC5965765 29769191

[5] Hoshi M , Oebisu N , Iwai T , et al. 2018 An usual presentation of pneumothorax associated with cystic metastasis from epithelioid sarcoma: a case report and review of the literature. Oncol. Lett. 15:4531–4534. 29541222 10.3892/ol.2018.7868 PMC5835908

